# Effects of dual-task training on chronic ankle instability: a systematic review and meta-analysis

**DOI:** 10.1186/s12891-023-06944-3

**Published:** 2023-10-13

**Authors:** Lu Wang, Ge Yu, Yaping Chen

**Affiliations:** Department of Rehabilitation, Beijing Tongren Hospital, Capital Medical University, 1 Dongjiaominxiang, Beijing, 100730 China

**Keywords:** Dual-task, Chronic ankle instability, Systematic review, Meta-analysis

## Abstract

**Background:**

Dual-task training has been a popular intervention for individuals with balance impairments. However, the effects of dual-task training on chronic ankle instability (CAI) have not been comprehensively analyzed and reliable clinical evidence is scarce. The purpose of this systematic review and meta-analysis is to evaluate the effectiveness of dual-task training on postural stability and functional ability in individuals with CAI.

**Methods:**

PubMed, Web of Science, EBSCO, Cochrane Library, Physiotherapy Evidence Database (PEDro), and China National Knowledge Infrastructure (CNKI) were researched from inception to November 2022. This study was conducted by the Preferred Reporting Items for Systematic Reviews and Meta-Analyses (PRISMA) guidelines. Two reviewers assessed the studies for inclusion and extracted data. The Cochrane Risk of Bias list was used to assess the risk of bias in included studies. Mean differences (MD) with a 95% confidence interval (CI) were calculated with the RevMan 5.3 software.

**Results:**

A total of 7 randomized controlled trials with 192 CAI met the inclusion criteria. The meta-analysis results showed that compared with the control group, dual-task training significantly improved the Y-balance test (MD = 1.60, 95% CI: −0.00 to 3.21, P = 0.050) and reduced COP-area (MD = − 0.94, 95% CI: −1.62 to − 0.26, P = 0.007) in individuals with CAI. However, there is no significant difference between dual-task training and the control group on COP-velocity (MD = − 0.26, 95% CI: −0.70 to 0.17, P = 0.240), hop test (MD = − 0.20, 95% CI: −0.66 to 0.26, P = 0.386) and BESS (MD = − 1.24, 95% CI: −2.95 to 0.48, P = 0.157) in individuals with CAI.

**Conclusion:**

This meta-analysis showed that dual-task training may be effective in improving static and dynamic postural stability. However, more high-quality randomized controlled trials are needed to verify the short and long-term effectiveness of dual-task training on CAI.

**Supplementary Information:**

The online version contains supplementary material available at 10.1186/s12891-023-06944-3.

## Introduction

Chronic ankle instability (CAI) is categorized into functional (FAI) and mechanical (MAI) instability and is characterized by symptoms of giving way, pain, and recurrent sprains. Following their first ankle sprain, 40% of people will develop CAI, which can result in static or dynamic postural instability and dysfunction [1]. Both automatic and cognitive control are involved in the process of postural stability and functional ability [2]. According to research, any reduction in conscious-controlled attention toward postural control increases the possibility of disturbing coordination and stability, presumably as a result of movement-specific reinvestment. Based on the notion of reinvestment, control movement performance might be affected by attention distraction [3]. Meanwhile, a theory based on the competition for cross-domain resources hypothesizes that limited cognitive resources are available for the management of maintaining postural stability and performing cognitive tasks, potentially resulting in a decline in postural stability, cognitive task performance, or both, when the two tasks are performed at the same time [4–6]. The constrained action hypothesis postulates that attentional shifts might enable motor systems to operate automatically, leading to more efficient performance [7]. Some studies [8, 9] found that dual-task training improved balance and functional performance in CAI. Nevertheless, no significant differential effect of dual-task training was observed in the study by Taghavi et al. [10]. Previous review studies indicated that dual-task training is safe and beneficial for postural stability in stroke patients and elderly individuals [11–13]. However, the effects of dual-task training on postural stability and functional ability in CAI have not been comprehensively analyzed, and reliable clinical evidence is scarce. Assessing whether dual-task training affects stability and functional ability may modify rehabilitation paradigms for CAI. We therefore performed a systematic review and meta-analysis of studies published on this individual. Our results can help healthcare providers decide whether dual-task training should be included in the rehabilitation program for CAI.

The purpose of this systematic review and meta-analysis was to investigate the effect of dual-task training on postural stability and functional ability in individuals with CAI.

## Methods

### Protocol and registration

This meta-analysis met the guidelines provided by the Preferred Reporting Items for Systematic Reviews and Meta-Analyses (PRISMA) [14]. This study was registered on PROSPERO as CRD42022356421. To increase the reliability of the meta-analysis results, only the randomized controlled trials (RCTs) were included and the effects of dual-task training on CAI were investigated, which were modified from the registered protocol.

### Search strategy

The search was performed in PubMed, Web of Science, EBSCO, Cochrane Library, Physiotherapy Evidence Database (PEDro), China National Knowledge Infrastructure (CNKI) from inception to November 2022 with no restriction on language. The search strategy for each database is shown in Appendix 1. The search terms used were chronic ankle instability OR functional ankle instability OR mechanical ankle instability OR ankle sprain OR ankle instability AND dual task OR cognitive OR motor OR divided attention OR multi task OR combined OR concurrent. We also screened the reference lists of the papers identified in database searches.

### Inclusion and exclusion criteria

The inclusion criteria were as follows: (1) individuals: individuals with chronic ankle instability; (2) intervention: dual-task training; (3) comparisons: no restrictions; (4) outcome measures: static and dynamic postural stability or functional tests; (5) study design: randomized controlled trials (RCTs). We excluded studies: (1) if they were conducted in animals, in vitro, cadavers, or simulators; (2) if the articles were not RCTs or not published as peer-reviewed journal articles, including book chapters and conference abstracts; (3) studies whose full text or data are not available.

### Study selection

Titles, abstracts, and full texts of the retrieved studies were screened by two independent reviewers (LW and GY) based on the inclusion and exclusion criteria. Disagreements were resolved by a third reviewer (YPC).

### Data extraction

Two researchers (LW and GY) independently extracted the data: characteristics of the publications (authors, year of publication, etc.); details of study design (sample size, etc.); individuals (age, gender, number of individuals, etc.); interventions (type of dual-task training, frequency, duration of the session, etc.); and outcomes (assessment methods, etc.). Disagreements were resolved by a third reviewer (YPC).

### Risk of bias assessment

The risk of bias of the included studies was assessed independently by two researchers (LW and GY) according to the Cochrane Risk of Bias list. The associated risks were divided into unclear, low, and high. Any disagreement was resolved by discussion until consensus was reached or by consulting a third author (YPC).

### Statistical analysis

Review Manager5.3 (Cochrane Collaboration, Oxford, UK) was used to analyze the outcomes. Continuous data were presented as mean difference (MD) and 95% confidence interval (95% CI). The I^2^ statistic was used to measure the heterogeneity across the included studies. The fixed effect model was applied to analyze data with low heterogeneity (P ≥ 0.1, I²<50%). For data with high heterogeneity (P < 0.1, I²>50%), the source of the heterogeneity was investigated. After the reduction of heterogeneity by subgroup analysis or sensitivity analysis, the fixed effect model was adopted. While the random effect model was used to analyze data with high heterogeneity. Publication bias was not performed as there were no more than 10 included studies for each outcome. P ≤ 0.05 was considered significant.

## Results

### Study identification

**Fig. 1:**
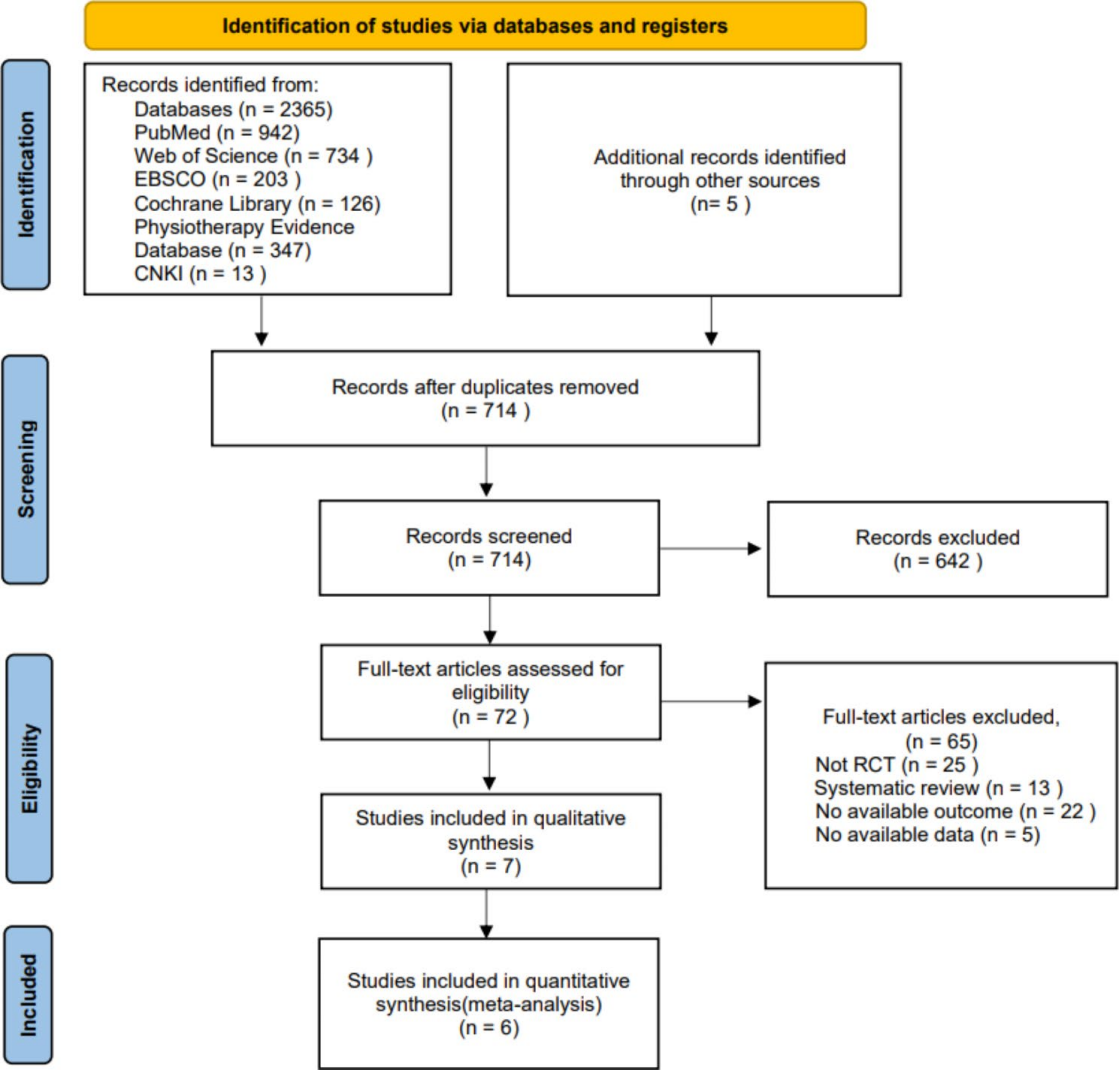
Flow diagram showing selection process of articles following PRISMA guidelines

A total of 72 articles were identified after duplicates were removed and titles and abstracts were screened from the initial search result (Fig. 1). In the end, seven studies [8–10, 15–18] with 192 individuals were included in this systematic review. Of which, six studies [9, 10, 15–18] were included in the meta-analysis statistical comparison. One study [8] was excluded from the meta-analysis because their outcome measurements could not be merged.

### Characteristics of included studies

The characteristics of the included studies are shown in Table 1. The five RCTs included a total of 54 FAI and 138 CAI. Five studies performed the single task with the balance training [8, 10, 15, 16, 18], one study with the proprioceptive exercises [9], and one study [17] with no intervention in the control group. The dual-task training performed with dual tasks of backwards counting (BC) [10, 15], random number generation (RNG) [8, 15], backward digit span task [9], catching thrown balls [16], kicking balls [17] and active video games [18]. The duration of the intervention ranged from 1 session to 5 weeks. No follow-up effect was investigated in the included studies.

**Table 1:** Characteristics of eligible studies included in the meta-analysis

Study	Design	Individual	Age (Years) EG/CG	Intervention			Outcome		Results and Conclusion
				Control	Dual-task training	Duration	Assessment tool	Outcome measure	
2022 Taghavi	RCT	21 CAI	18–25 22.42 ± 2.22/23.14 ± 1.34	Balance training	Balance training + BC	50 min/session, 3 sessions/week, 5 weeks	Force plate	Y-balance test, BESS	Although the group with CI showed a greater improvement in mean than group without CI, but the difference was not significant in any of the variables.
2020 Onegh	RCT	34 FAI	18–52 34.82 ± 11.18/31.06 ± 9.90	Balance training	Balance training + RNG	30 min/session, 3 sessions/week, 4 weeks	Biodex balance system	OSI, APSI, MLSI	There were no differences between the two groups regarding the study variables. Dual-task training improved more balance indices compared to the control group. It is recommended that dual-task training be included in a physiotherapy program for people with FAI.
2020 Chae	RCT	30 CAI	>18 35.33 ± 7.05/34.27 ± 6.77	Proprioceptive exercises	Proprioceptive exercises + Backward digit span task	15 min/session, 3 sessions/week, 4 weeks	Force plate	COP, Y-balance test, Side hop, Figure-of-8 hop, Square hop test	The experimental group showed more significant improvement than the controls in terms of the fluctuation distance, speed, and area of static balance.
2017 Gonzales	RCT	23 CAI	18–35 20.20 ± 1.10/20.73 ± 1.10	Balance training	Balance training + RNG/BC	20-30 min/session, 14 sessions, 4 weeks	Force plate	Y-balance test, TTB, COP	Dual tasks have the potential to influence training as demonstrated by the moderate to strong effects it had on TTB and Y-balance outcomes.
2016 Kwak	RCT	20 FAI	16–25 18.50 ± 4.80/17.80 ± 1.80	Balance training	Balance training + Catching thrown balls	30 min/day, 3 days/week, 4 weeks	Foot scan, Tech-Storm Co.	Anterior-posterior, Medio-lateral balance, Up-down hop, Figure-of-8 hop, Single hop test	Dual-task training improved balance and functional performance better than single-task training.
2016 Conceição	RCT	44 CAI	24.00 ± 4.00/ 22.00 ± 3.00	No intervention	Balance training + Kicking balls	30 min/session, 1 session	Force plate	COP	A single session of ball-kicking balance-perturbation training promoted changes in postural-control strategies in individuals with CAI.
2014 Maresh	RCT	20 CAI	21.2 ± 2.5/ 21.2 ± 2.5	Balance training	Balance training + Active video games	15-25 min/session, 3–5 sessions/week, 4 weeks	Force plate	Y-balance test, BESS	No significant difference between groups.

RCT: Randomized Controlled Trails; CAI: Chronic Ankle Instability; FAI: Functional Ankle Instability; EG: Experimental Group; CG: Control group; BESS: Balance Error Scoring System; OSI: Overall Stability Index; APSI: Anterior-posterior Stability Index; MLSI: Medial-lateral Indices Stability Index; COP: Center of pressure; TTB: Time to Boundary; BC: Backwards Counting; RNG: Random Number Generation

### Study bias

**Fig. 2:**
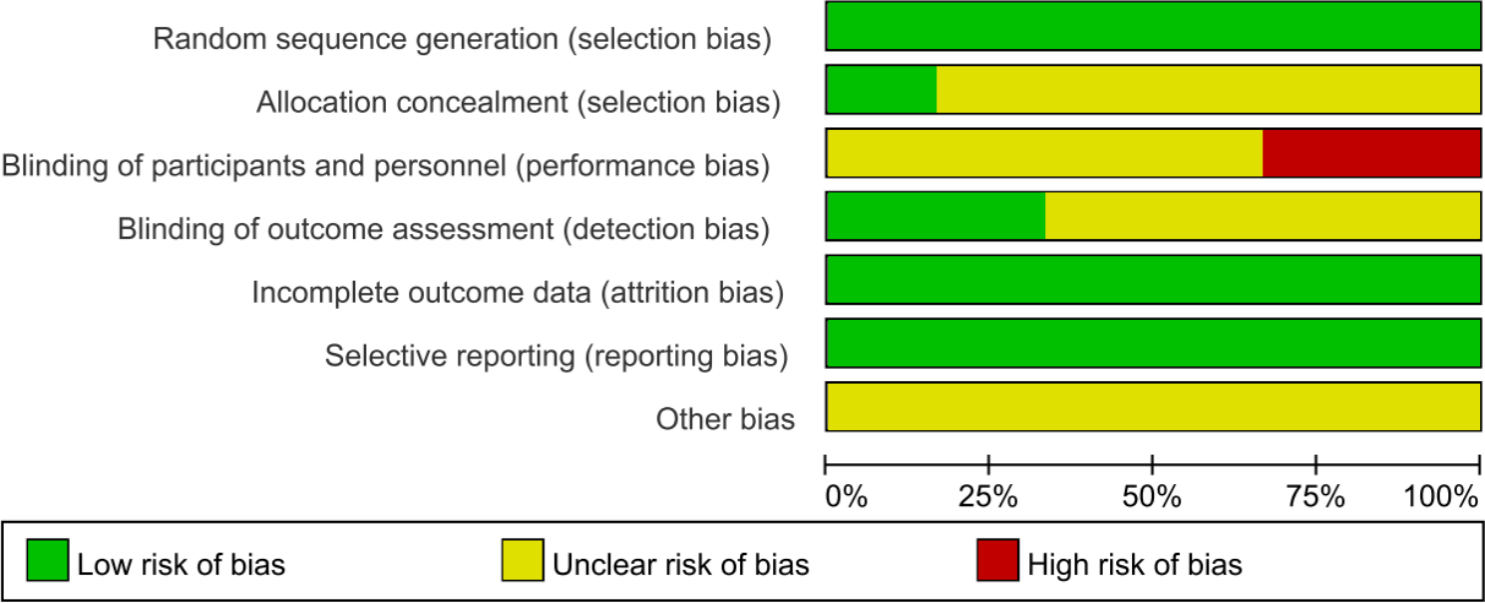
Risk of bias graph

**Fig. 3:**
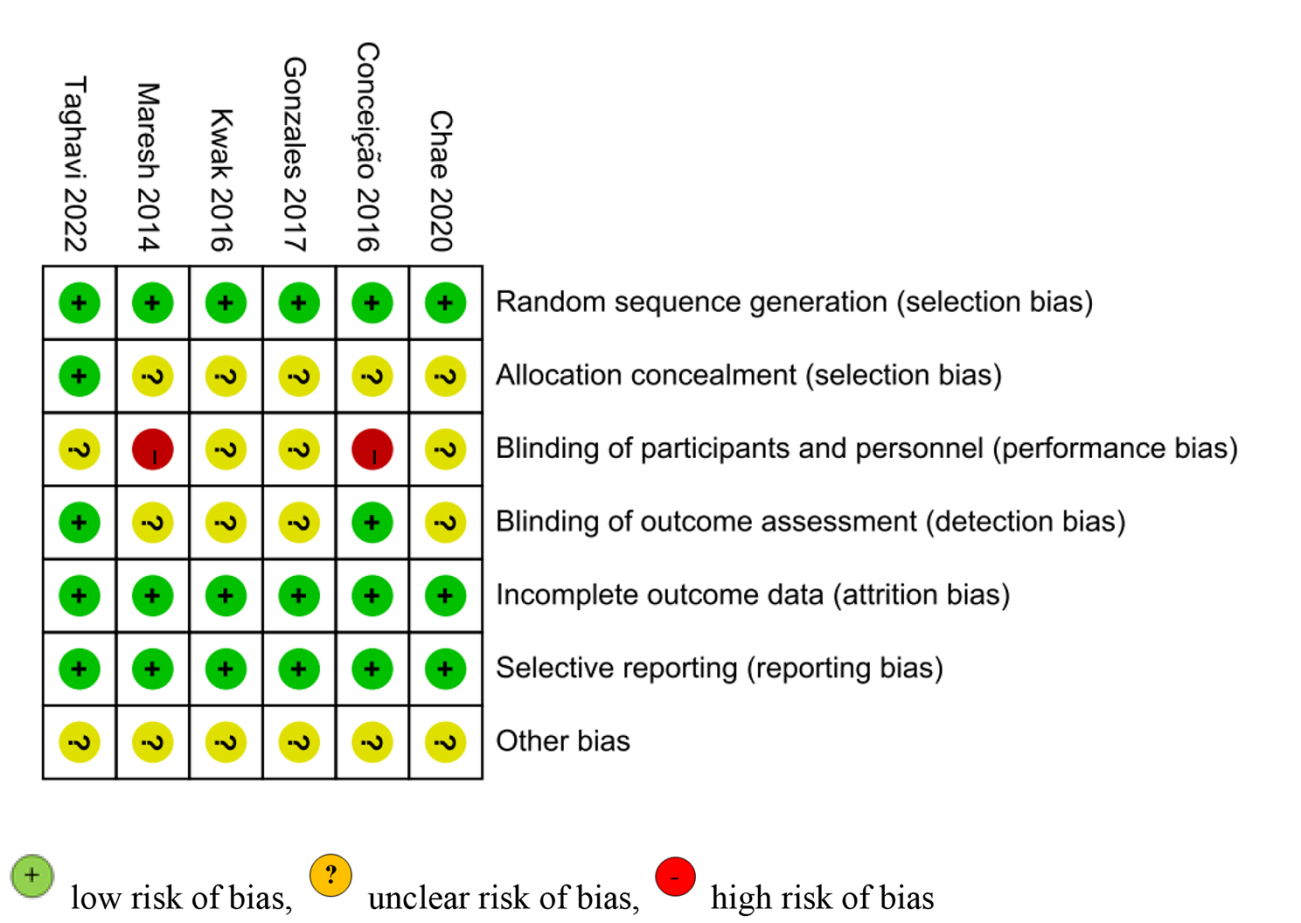
Risk of bias summary

The bias of the graph and summary of the included studies are shown in Figs. 2 and 3.

Blinding of individuals and personnel in 2 studies [17, 18] was considered high-risk. In 2 studies [10, 17], the risk of bias associated with blinding outcome assessment was deemed low. In 1 study [10], the risk of bias from allocation concealment was found to be low. Other bias in the included studies was deemed unclear.

### Synthesis of results

**Fig. 4:**
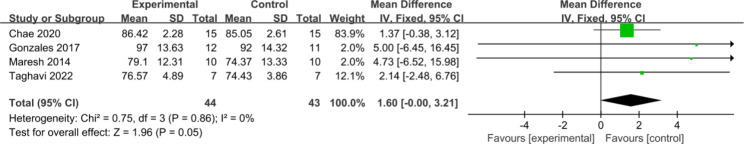
Forest plots demonstrated the effect of dual-task training on Y-balance in individuals with CAI.

Four studies [9, 10, 15, 18] with 87 individuals reported the effects of dual-task training on the Y-balance test. A fixed-effect model was used (I^2^ = 0%, P = 0.860). The synthesized data showed marginally significant differences between dual-task training and single-task training in Y-balance (MD = 1.60, 95% CI: −0.00 to 3.21, P = 0.050). (Fig. 4).

**Fig. 5:**
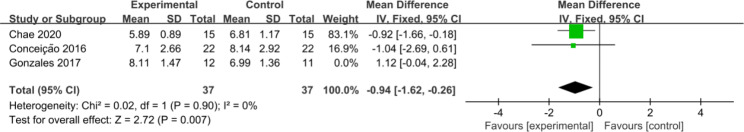
Forest plots demonstrated the effect of dual-task training on COP-area in individuals with CAI.

Three studies [9, 15, 17] with 97 individuals reported the effects of dual-task training on center of pressure-area (COP-area) with high heterogeneity (I^2^ = 78%, P = 0.011). After excluding one study [15] by sensitivity analysis, heterogeneity among the studies was small (I^2^ = 0%, P = 0.897). A fixed-effect model was used for analysis, and the synthesized data showed a significant decrease in COP-are in the dual-task training group (MD = − 0.94, 95% CI: −1.62 to − 0.26, P = 0.007) in CAI. (Fig. 5).

**Fig. 6:**
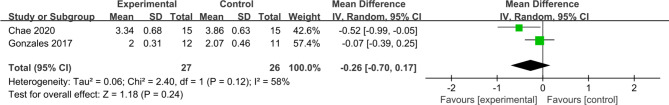
Forest plots demonstrated the effect of dual-task training on COP-velocity in individuals with CAI.

Two studies [9, 15] with 53 individuals reported the effects of dual-task training on COP- velocity. The synthesized data showed no significant differences between dual-task training and single-task training in COP-velocity (MD = − 0.26, 95% CI: −0.70 to 0.17, P = 0.240) in CAI. And a random-effect model was used because of substantial heterogeneity for COP-velocity (I^2^ = 58%, P = 0.122) (Fig. 6).

**Fig. 7:**
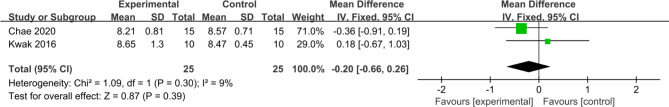
Forest plots demonstrated the effect of dual-task training on figure-of-8 hop tests in individuals with CAI.

Two studies [9, 16] with 50 individuals reported the effects of dual-task training on figure-of-8 hop test in CAI. No significant difference was found between dual-task training and single-task training (MD = − 0.20, 95% CI: −0.66 to 0.26, P = 0.386). A fixed-effect model was used (I^2^ = 9%, P = 0.296) (Fig. 7).

**Fig. 8:**
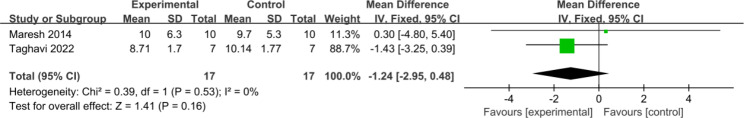
Forest plots demonstrated the effect of dual-task training on BESS scores in individuals with CAI.

Two studies [10, 18] with 34 individuals reported the effects of dual-task training on balance error scoring system (BESS) score in CAI. No significant difference was found between dual-task training and single-task training (MD = − 1.24, 95% CI: −2.95 to 0.48, P = 0.157). A fixed-effect model was used (I^2^ = 0%, P = 0.531) (Fig. 8).

## Discussion

This meta-analysis summarizes the effects of dual-task training on postural stability and functional ability among CAI groups. The results showed that compared with single task training or no intervention, dual-task training can improve Y-balance and COP-area in individuals with CAI.

### Y-balance test

This meta-analysis showed that dual-task training had a marginally significant advantage over the control group on the Y-balance test in CAI. Similar results were obtained for dynamic balance performance in older adults [19] and in multiple sclerosis [20]. Several studies have shown that the process controlling the postural changes required to maintain stability requires attention [21, 22]. In the CAI group, a higher level of gait disturbance was reported to be required to cause a change, such as reducing stride time variability in walking, compared to healthy individuals, which may indicate lower adaptability of the sensorimotor system, reducing the ability of the central nervous system to adjust to different task demands [23]. Higher ankle inversion and frontal plane movement variability were detected in the FAI group compared to the healthy controls group during cognitive load walking, which may enhance the risk of ankle instability. Furthermore, the considerably lower mean stride velocity and cognitive performance in both groups imply that walking requires attention, and central processing requires attention to collect and integrate sensory information [24]. The cross-domain resource competition hypothesis states that both maintenances of postural stability and performance on cognitive tasks draw from a finite pool of cognitive resources for their regulation, potentially leading to a decrease in postural stability and/or cognitive tasks performance, when the two activities are performed simultaneously [25]. Nevertheless, some studies [26–29] examining individuals with CAI have shown positive effects of using two tasks, including improvements in postural control. The authors hypothesized that this could be due to increased attention to postural control [27]. Another study [30] confirmed the benefits of dual-task training for the task integration hypothesis, which states that task coordination skills improve when two activities are practiced simultaneously. Similar benefits were shown for variable priority training compared to fixed priority training in a study by Kramer et al. [31], which also demonstrated that individuals can learn to coordinate between two activities during training under variable priority settings. In addition, one study [32] found radiological evidence that cerebral hemodynamics of the dorsolateral prefrontal cortex improved in the dual-task training group, which was related to the increase in performance. However, some studies showed no significant difference between the dual-task training group and the control group in the dynamic postural control of stability indices [8] and Y-balance test [9, 10, 15, 18] in individuals with CAI. This may be due to the different intervention protocols included in the studies.

### COP

The meta-analysis showed that dual-task training had a significant advantage over the control group in COP-area. It is consistent with previous studies, which found that a dual-task training group significantly improved static balance of anterior-posterior and medio-lateral balance [16] in CAI. During dual-task, individuals need to divide attention between two tasks (capacity sharing theory) [33]. Improvements in attention by dual-task training could affect balance [34]. In addition, dual-task training might improve integration and coordination skills while performing two tasks simultaneously [35]. However, there is no significant difference between dual-task training group and the control group on COP-velocity in CAI. The choice of outcome measurement may influence the results of the study since youths demonstrated low reliability in terms of COP-velocity [36]. The selection of more sensitive indices could make this assessment more accurate. Population may also be the reason for the negative results in this study. The sensitivity to dual-task training increased in the elderly and neurological diseases [37–39]. CAI population with younger age and better health conditions might have better adaptive capacity to dual tasks. The heterogeneity in COP-are and COP-velocity may be explained by the different use of dual-task training like the backward digit span task [9], RNG plus BC [15] and kicking balls [17]. Further research suggested that the degree of difficulty of the postural task and the complexity of the dual tasks both had an impact on stability performance [12, 40]. When the cognitive task is more complicated, more attentional resources may be required, depleting the resources available for postural stability. With increased balancing difficulty, attentional demand for postural control has been found to rise [4]. Even relatively straightforward dual tasks may have a detrimental effect on postural stability [11].

### Hop-test

According to this meta-analysis, the dual-task training group had no significant advantage over the control group on the hop test in CAI, supporting previous findings [9, 41]. During dual-task, postural control appeared to take priority over cognitive processing [42]. It is unlikely that the cognitive task will have any impact on the postural control test if it is insufficient [43]. And it is interesting to note that, given the fact that the intervention program comprised dual tasks, we might have found higher improvement in the dual-task training group compared to the balance training group if the evaluation method had been dual-task [8]. Nevertheless, Kwak et al. [16] found that the dual-task training group improved significantly more than the control group in the up-down hop test. In this study, the motor task was part of the dual task. Motor task training may be more effective than cognitive task training for enhancing postural control, because motor tasks like throwing, catching, and kicking involve complex interactions of the somatosensory, visual, and vestibular systems to manage relationships between the body and external environment [44]. Further research is needed to confirm this.

### BESS

BESS is a reliable and inexpensive tool for measuring the static balance of standing in people with CAI. Based on this meta-analysis, dual-task training had no significant effect over the control group on BESS in CAI. This is confirmed by a previous study [45], which stated that performing cognitive tasks with balance training simultaneously did not improve static balance while standing compared to balance training alone. Attentional demands are closely associated with postural control [46]. Quiet standing, which requires less attention because it is a basic ability that individuals have mastered [29], could explain why there is no significant difference in BESS between dual-task training and the control group in CAI.

### Limitations of the study

There are several limitations in this study. (1) The included studies varied in the type and degree of difficulty of dual-task training, which added to the heterogeneity. (2) The duration of dual-task training’s therapeutic impact on CAI is unknown due to the absence of follow-up investigations. (3) Due to the small number of RCTs and limited studies in each category after sensitivity analysis, there was a potential risk of bias. (4) The majority of the studies that were included had small sample sizes, which increases the likelihood of a type II error [47].

### Future perspectives

Maintaining posture is a simultaneous task in a variety of daily living activities, athletic pursuits, and leisure activities. Training protocols for individuals with CAI should be developed as dual-task training in the future to help the sensorimotor system become more automatic and perform its activities subconsciously [10]. Additionally, balance training strategies that divert attention away from postural control (i.e., external focus of attention) are more successful than those that concentrate on postural control (i.e., internal focus of attention) [48, 49]. Balance protocols that shift focus away from postural control are recommended in future studies. Besides, we might have found higher improvement in the dual-task training group compared to the balance-training group if the evaluation method had been dual-task. Outcome measurements involving dual-task performance were suggested to assess the effects of dual-task training on individuals.

## Conclusion

Dual-task training may be effective in improving static and dynamic postural stability. However, more high-quality randomized controlled trials are needed to verify the short and long-term effectiveness of dual-task training on CAI.

### Electronic supplementary material

Below is the link to the electronic supplementary material.

Supplementary Material 1

Supplementary Material 2

## Data Availability

The datasets used and/or analysed during the current study available from the corresponding author on reasonable request.

## Abbreviations

CAI: Chronic ankle instability
FAI: Functional ankle instability
MAI: Mechanical ankle instability
PEDro: Physiotherapy evidence database
CNKI: China national knowledge infrastructure
PRISMA: Preferred reporting items for systematic reviews and meta-analyses
MD: Mean differences
CI: Confidence Interval
RCTs: Randomized controlled trials
BC: Backwards counting
RNG: Random number generation
COP: Center of pressure
BESS: Balance error scoring system
EG: Experimental group
CG: Control group
OSI: Overall stability index
APSI: Anterior-posterior stability index
MLSI: Medial-lateral indices stability index
TTB: Time to boundary
