# Supplementary material for: Effects of dual-task training on chronic ankle instability: a systematic review and meta-analysis
Source: BMC Musculoskelet Disord. 2023 Oct 13;24:814. doi: 10.1186/s12891-023-06944-3 (PMC10571247; doi:10.1186/s12891-023-06944-3)
Supplement: Supplementary file 1 — Supplementary Material 1 [file 12891_2023_6944_MOESM1_ESM.docx]

**Appendix 1**

Search Strategies for PubMed, Web of Science, EBSCO, Cochrane Library, Physiotherapy Evidence Database (PEDro), and China National Knowledge Infrastructure (CNKI). We included papers published from their inception to November 2022 with no restriction on language.

**1. PubMed**

#1 “chronic ankle instability” [Title/Abstract] OR “functional ankle instability” [Title/Abstract] OR “mechanical ankle instability” [Title/Abstract] OR “ankle sprain” [Title/Abstract] OR “ankle instability” [Title/Abstract]

#2 “dual task” [Title/Abstract] OR “cognitive” [Title/Abstract] OR “motor” [Title/Abstract] OR “divided attention” [Title/Abstract] OR “multi task” [Title/Abstract] OR “combined” [Title/Abstract] OR “concurrent” [Title/Abstract]

#3 #1 AND #2

**2. Web of Science**

#1 TS= (“chronic ankle instability” OR “functional ankle instability” OR “mechanical ankle instability” OR “ankle sprain” OR “ankle instability”)

#2 TS= (“dual task” OR “cognitive” OR “motor” OR “divided attention” OR “multi task” OR “combined” OR “concurrent”)

#3 #1 AND #2

**3. EBSCO**

S1 TX (“chronic ankle instability” OR “functional ankle instability” OR “mechanical ankle instability” OR “ankle sprain” OR “ankle instability”)

S2 TX (“dual task” OR “cognitive” OR “motor” OR “divided attention” OR “multi task” OR “combined” OR “concurrent”)

S3 S1 AND S2

**4. Cochrane Library**

#1 “chronic ankle instability” OR “functional ankle instability” OR “mechanical ankle instability” OR “ankle sprain” OR “ankle instability”: ti,ab,kw

#2 “dual task” OR “cognitive” OR “motor” OR “divided attention” OR “multi task” OR “combined” OR “concurrent”: ti,ab,kw

#3 #1 AND #2

**5. Physiotherapy Evidence Database (PEDro)**

Abstract & Title: “ankle instability”

Title Only: “dual task”

Match any search term (OR)

**6. China National Knowledge Infrastructure (CNKI)**

(SU='双重任务' OR SU='多重任务' OR SU='认知' OR SU='运动' OR SU='注意力') AND (SU='踝关节不稳' OR SU='踝关节损伤')
